# The Effects of Aging on the Regulation of T-Tubular *I*_Ca_ by Caveolin in Mouse Ventricular Myocytes

**DOI:** 10.1093/gerona/glx242

**Published:** 2017-12-09

**Authors:** Cherrie H T Kong, Simon M Bryant, Judy J Watson, Hanne C Gadeberg, David M Roth, Hemal H Patel, Mark B Cannell, Clive H Orchard, Andrew F James

**Affiliations:** 1 School of Physiology, Pharmacology & Neuroscience, University of Bristol, UK; 2 VA San Diego Healthcare System and Department of Anesthesiology, University of California, San Diego

**Keywords:** Caveolin-3, Excitation-contraction coupling, Ca signaling

## Abstract

Aging is associated with diminished cardiac function in males. Cardiac excitation-contraction coupling in ventricular myocytes involves Ca influx via the Ca current (*I*_Ca_) and Ca release from the sarcoplasmic reticulum, which occur predominantly at t-tubules. Caveolin-3 regulates t-tubular *I*_Ca_, partly through protein kinase A (PKA), and both *I*_Ca_ and caveolin-3 decrease with age. We therefore investigated *I*_Ca_ and t-tubule structure and function in cardiomyocytes from male wild-type (WT) and caveolin-3-overexpressing (Cav-3OE) mice at 3 and 24 months of age. In WT cardiomyocytes, t-tubular *I*_Ca_-density was reduced by ~50% with age while surface *I*_Ca_ density was unchanged. Although regulation by PKA was unaffected by age, inhibition of caveolin-3-binding reduced t-tubular *I*_Ca_ at 3 months, but not at 24 months. While Cav-3OE increased cardiac caveolin-3 protein expression ~2.5-fold at both ages, the age-dependent reduction in caveolin-3 (WT ~35%) was preserved in transgenic mice. Overexpression of caveolin-3 reduced t-tubular *I*_Ca_ density at 3 months but prevented further *I*_Ca_ loss with age. Measurement of Ca release at the t-tubules revealed that the triggering of local Ca release by t-tubular *I*_Ca_ was unaffected by age. In conclusion, the data suggest that the reduction in *I*_Ca_ density with age is associated with the loss of a caveolin-3-dependent mechanism that augments t-tubular *I*_Ca_ density.

It is generally recognized that aging is associated with changes in normal cardiac function, although the cellular mechanisms underlying this remodeling remain unclear (1,2). It is becoming apparent that the effects of age on the heart differ between the sexes (2). For example, while the contractile amplitude of ventricular myocytes isolated from male mouse hearts were reduced by age, age did not affect contractility of myocytes from female mouse hearts (3,4). In male ventricular myocytes, reduced L-type Ca current (*I*_Ca_) density (4–7), altered ryanodine receptor (RyR) activity and slowed sarcoplasmic reticulum (SR) Ca uptake have been suggested to contribute to the effects of physiological aging on excitation-contraction (E-C) coupling (8–11). Transverse (t-) tubules, invaginations of the surface membrane that are central to E-C coupling (12–14), are known to be labile (15), and changes in both t-tubule structure and function have been implicated in the impaired contractility observed in heart failure (16,17). However, the effect of aging on t-tubule structure and function is unknown.

The cholesterol-binding membrane protein caveolin-3 (Cav-3) has been suggested to contribute to t-tubule development (18,19) and also plays an important role in the localization of a striking variety of ion channels, transporters, and signaling proteins at the sarcolemma of cardiac myocytes (20–22), including the localization of L-type Ca channels (LTCCs; and thus *I*_Ca_), Na-Ca exchange (NCX) and *β*_2_-adrenoceptors, to the t-tubules (23–27). It has also been suggested that Cav-3 plays a role in the constitutive regulation of *I*_Ca_ at the t-tubules (25). Recent studies have shown that Cav-3 expression declines with age (28,29) and a role has been suggested for this decrease in Cav-3 expression in the development of the aged phenotype (30).

We have, therefore, investigated the effect of age on t-tubule structure, *I*_Ca_, and intracellular Ca transients, in myocytes from male wild-type (WT) mice and whether cardiac-specific over-expression of Cav-3 (31) protects against the effects of aging on the heart.

## Methods

Further details of experimental methods are provided in the Supplementary Material available online (https://academic.oup.com/biomedgerontology).

### Animals

All procedures were performed in accordance with UK legislation. Transgenic mice with cardiac myocyte-specific over-expression of Cav-3 (Cav-3OE) were generated using animals from Tsutsumi et al. (31) and WT C57Bl/6 littermates. Animals were kept in temperature-controlled rooms with ad libitum access to food and water.

### Myocyte Isolation

Ventricular myocytes were isolated from the hearts of 3- and 24-month-old male WT and Cav-3OE mice. Animals were injected with heparin (500 I.U., i.p.) and 5 minutes later killed by cervical dislocation, the heart rapidly excised and myocytes isolated using our standard methods (26), and used on the day of isolation.

### Solutions

The standard perfusion solution used in these experiments contained (in mM): 133 NaCl, 5 KCl, 1 MgSO_4_, 1 CaCl_2_, 1 Na_2_HPO_4_, 10 D-glucose, 10 4-(2-hydroxyethyl)-1-piperazineethanesulfonic acid (HEPES), pH 7.4 (NaOH). During electrophysiological recordings, KCl was substituted with CsCl to inhibit K currents and the pipette solution contained (in mM): 110 CsCl, 20 TEACl, 0.5 MgCl_2_, 5 MgATP, 5 BAPTA, 10 HEPES, 0.4 GTP-Tris, pH 7.2 (CsOH). All experiments were performed at room temperature. Where stated, 1 μM of TAT-tagged Cav-3 scaffolding domain (C3SD) peptide (32,33) was used as described previously (25). The C3SD peptide is thought to disrupt binding of Cav-3 to its partner proteins at the scaffolding domain (32,33). While the role of the caveolin scaffolding domain in interactions with partner proteins has been questioned (34,35), pretreatment of cardiac myocytes with the peptide has previously been shown to inhibit Cav-3-dependent signaling compared with cells treated with scrambled control peptide (25,32,33).

### Statistics

Data are expressed as mean ± SEM. Paired and unpaired *t* tests or Mann–Whitney test and one- or two-way analysis of variance (ANOVA) were used with the Bonferroni post-hoc test where applicable. Current density-voltage relationship curves were analyzed with two-way repeated measures (RM) ANOVA with Bonferroni post-hoc test. The limit of statistical confidence was *p* < .05. Sample sizes (*n*/*N*) represent the numbers of cells and animals, respectively.

## Results

### Effect of Age and Cav-3OE on Cell Morphology

**Figure 1.**
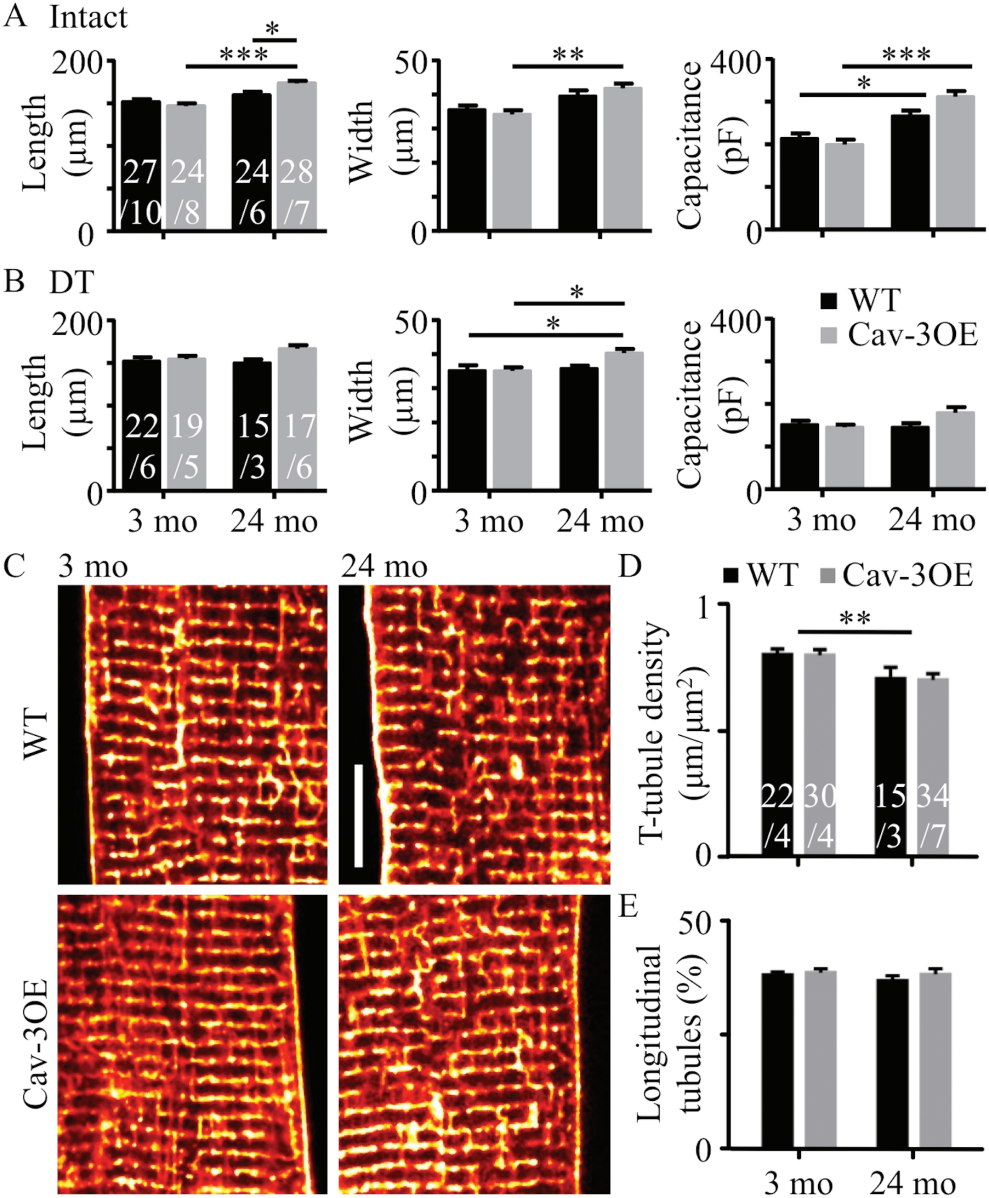
Effect of age and Cav-3 over-expression on cell size and t-tubule organization. (**A**) Mean cell length, width and capacitance measured in intact myocytes from wild-type (WT) (black bars) and Cav-3OE (gray bars) mice at 3 months and 24 months of age. Two-way analysis of variance (ANOVA) (age, genotype) tests yielded results as follows. Length: age *p* < .001, genotype *ns*, interaction *p* < .01. Width: age *p* < .001, genotype *ns*, interaction *ns*. Capacitance: age *p* < .001, genotype *ns*, interaction *p* < .05. (**B**) Corresponding data from detubulated (DT) myocytes. Length: age *ns*, genotype *p* = .03, interaction *ns*. Width: age *p* = .027, genotype *ns*, interaction *ns*. Capacitance: age *ns*, genotype *ns*, interaction *p* = .049. Asterisks indicate *p < .05, **p < .01, and ***p < .001 (Bonferroni corrected post-hoc test). *n/N* indicated on bars. (**C**) Representative confocal images of t-tubules labeled with di-8-ANEPPs. Scale bar shows 10 μm. (**D**) Mean t-tubule skeleton density. (**E**) Mean percentage of t-tubules that were oriented along the long-axis of the cell (“longitudinal”). Asterisks and *n/N* as in **A** and **B**.

Aging from 3 to 24 months was associated with cellular hypertrophy in cardiac myocytes. Figure 1A shows mean data for length and width of myocytes isolated from WT and Cav-3OE mice at 3 and 24 months of age. Age was associated with an increase in cell length (*p* < .001, two-way ANOVA) and cell width (*p* < .001, two-way ANOVA) with the increase in length and width being greater in the Cav-3OE than in WT mice (increase in length: WT ~5%, Cav-3OE ~19% and width: WT ~11%, Cav-3OE ~22%). Cell capacitance, an electrical measure of cell surface membrane area, also increased with age in both WT and Cav-3OE cells (by ~24% and 55%, respectively; *p* < .001, two-way ANOVA, Figure 1A). There was no difference in cell width between WT and Cav-3OE myocytes at either 3 or 24 months, whereas at 24 months, but not at 3 months, Cav-3OE myocytes were longer than WT (*p* < .05, two-way ANOVA, Bonferroni post hoc test). Mean cell width, length, and capacitance of detubulated cells are shown in Figure 1B.

The relationship between cell membrane area and cell size is difficult to predict due to the presence of t-tubules. We therefore constructed a simple geometric model cell to examine the expected relationship between membrane area and cell size, assuming no changes in t-tubule density (for details, see Supplementary Material). In brief, myocyte geometry was approximated by a closed elliptical cylinder, with t-tubules approximated by round cylinders invaginating the cell. The model predicted a 22% increase in total membrane area of WT myocytes and a 55% increase in total membrane area of Cav-3OE myocytes simply as a result of the measured age-dependent hypertrophy, which agrees well with the observed increases in cell capacitance with age in the two genotypes (24% and 55%, respectively).

To examine the effects of age and Cav-3OE on t-tubule structure, live myocytes were stained with di-8-ANEPPS to label lipid membranes continuous with the surface sarcolemma. Representative confocal images show modest changes in t-tubule organization with age (Figure 1C). Quantification of the t-tubule skeleton showed that aging in WT and OE myocytes was associated with a 12% and 14% reduction in t-tubule density (*p* < .01, two-way ANOVA), respectively (Figure 1D), with no significant effect of Cav-3OE. This slight decrease in t-tubule density with age was not accompanied by changes in tubule orientation, as the proportion of longitudinal tubules remained the same (Figure 1E). Cav-3OE did not appear to alter tubule orientation over this age range.

Taken together, these data suggest that aging is accompanied by an increase in cell width and capacitance, with a small decrease in t-tubule density. While the age-related hypertrophy was augmented slightly in Cav-3OE myocytes, Cav-3 over-expression had little effect on t-tubule morphology at either age and did not ameliorate the effect of age on cell and t-tubule morphology.

### Effect of Age and Cav-3OE on I_Ca_

**Figure 2.**
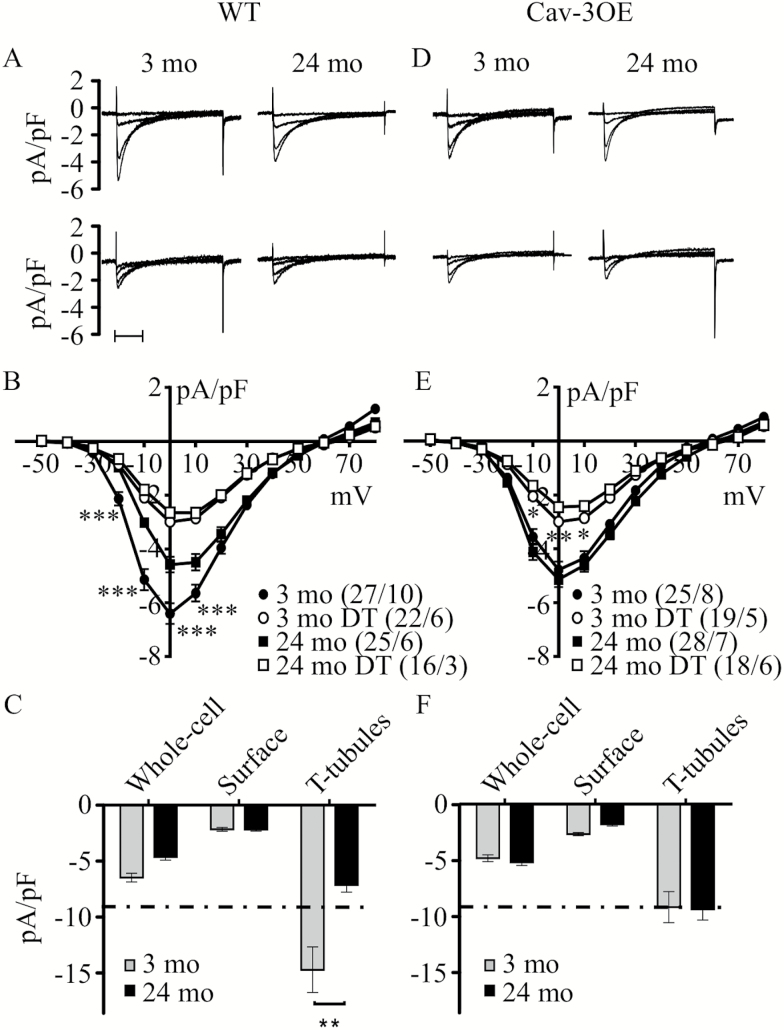
Effect of age and Cav-3 over-expression on *I*_Ca_ density. (**A**) Representative records of *I*_Ca_ elicited by step depolarizations to −30, −20, −10, and 0 mV recorded from intact (top panels) or detubulated (DT, bottom panels) myocytes isolated from 3- and 24-month-old wild-type (WT) mice. Scale bars show 100 ms. (**B**) Mean *I*_Ca_ density-voltage relationships recorded from 3-month intact (closed circles) and DT (open circles) myocytes, and 24-month intact (closed squares) and DT (open squares) myocytes from WT and Cav-3OE animals. Analysis with two-way repeated measures analysis of variance (RM ANOVA) yielded: intact myocytes, age *p* < .01, voltage *p* < .001, interaction *p* < .001; DT myocytes, age *ns*, voltage *p* < .001, interaction *ns*. (**C**) Mean *I*_Ca_ density at 0 mV for intact and DT (“surface”) myocytes, with estimated t-tubular *I*_Ca_ density in 3- (gray) and 24-month (black) WT myocytes. ** indicates *p* < .01 by Student’s *t* test. (**D**) Corresponding representative records of *I*_Ca_ intact and DT myocytes isolated from 3- and 24-month-old Cav-3OE mice, to the same time scale as panel **A**. (**E**) Mean *I*_Ca_ density-voltage relationships recorded from Cav-3OE myocytes, using the same key as panel **B**. Analysis with two-way RM ANOVA yielded: intact myocytes, age *ns*, voltage *p* < .001, interaction *ns*; DT myocytes, age *ns*, voltage *p* < .001, interaction *p* < .001. (**F**) Mean *I*_Ca_ density at 0 mV for whole cell and DT Cav-3OE myocytes, with estimated t-tubular *I*_Ca_ density. Dashed horizontal line in (**C**) and (**F**) corresponds to t-tubular *I*_Ca_ density of Cav-3OE myocytes (−9.3 pA/pF). **B** and **E**: *p < .05, **p < .01, and ***p < .001 (Bonferroni corrected post-hoc test), **C**: *p < .05 (Student’s *t* test).

Since Cav-3 has been implicated in localization of *I*_Ca_ to the t-tubules (25,27), we investigated *I*_Ca_ distribution and regulation with age and Cav-3 over-expression. *I*_Ca_ was recorded from intact (Figure 2A, top) and DT (Figure 2A, bottom) myocytes from 3-month (left panels) and 24-month (right panels) WT hearts. The corresponding *I*_Ca_ density-voltage relationships (Figure 2B) show that *I*_Ca_ density was reduced with age. Absolute *I*_Ca_ in WT myocytes was not significantly different at the two ages (Supplementary Table 1), which suggests that the decrease in *I*_Ca_ density with age was primarily due to the increase in membrane area (by 24%, measured as cell capacitance) without a commensurate increase in LTCC number. Assuming no change in absolute *I*_Ca_, either at the t-tubules or at the surface sarcolemma, the geometric model predicts that the increase in total membrane area due to cellular hypertrophy would be associated with a greater decrease in *I*_Ca_ density at the t-tubules than at the surface membrane (Supplementary Figure S1, G and H). To test this idea, *I*_Ca_ was recorded from DT 3-month and 24-month WT myocytes. *I*_Ca_ density was reduced following DT at the two ages, consistent with the predominant localization of *I*_Ca_ to the t-tubules but there was no significant difference in *I*_Ca_ density in DT cells at the two ages (Figure 2B; Supplementary Table S2). Thus, in WT myocytes, t-tubular *I*_Ca_ density was decreased by ~50% (*p* < .002, *t* test) while that at the cell surface was unchanged with age (Figure 2C). This compares with the 23% decrease in t-tubular *I*_Ca_ density and 15% decrease in surface sarcolemmal *I*_Ca_ density predicted by the model as a result of cellular hypertrophy alone. Thus, the data show that age was associated with a loss of *I*_Ca_ density from the t-tubules specifically, an effect that cannot be accounted for by cellular hypertrophy alone.

In intact Cav-3OE myocytes, both absolute *I*_Ca_ and cell capacitance increased with age. In consequence, unlike WT myocytes, *I*_Ca_ density in intact Cav-3OE myocytes was unchanged with age (two-way RM ANOVA, age *ns*, interaction *ns*; Figure 2D, E). In DT myocytes, cell capacitance and *I*_Ca_ density were not significantly different with age. Figure 2F shows calculated *I*_Ca_ density at the t-tubules, compared to that at the cell surface, and shows that unlike in WT myocytes, t-tubular *I*_Ca_ density in Cav-3OE myocytes was unchanged with age. This contrasts with the decrease predicted by the model on the basis of simple geometric considerations, and suggests maintenance of *I*_Ca_ as a result of Cav-3 OE.

These data also show that over-expression of Cav-3 has a different effect on *I*_Ca_ in 3-month and 24-month myocytes. *I*_Ca_ density in intact myocytes was reduced by over-expression of Cav-3 at 3 months but not at 24 months. Comparison of Figure 2C and F shows that the major effects of over-expression of Cav-3 were to decrease t-tubular *I*_Ca_ at 3 months, and inhibit further age-associated decrease in t-tubular (and thus, whole cell) *I*_Ca_ density, with little effect at the cell surface.

**Figure 3.**
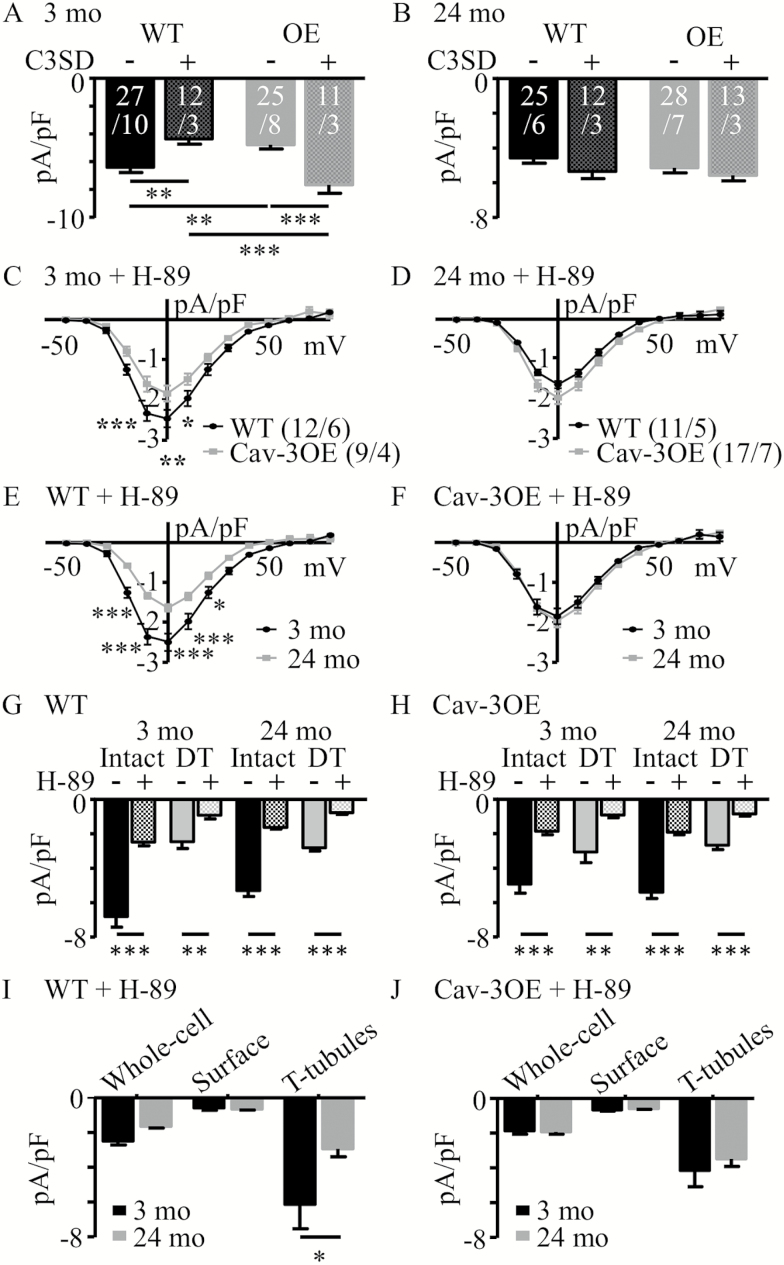
Effect of inhibition of Cav-3 and PKA on *I*_Ca_ density in 3- and 24-month myocytes. (**A**) Mean *I*_Ca_ density at 0 mV in the absence (−) and presence (+) of C3SD peptide in wild-type (WT) and Cav-3OE myocytes for myocytes from 3-month-old mice (two-way ANOVA: C3SD *ns*, genotype *ns*, interaction *p* < .001). (**B**) Corresponding data for 24-month-old mice (two-way ANOVA: C3SD *ns*, genotype *ns*, interaction *ns*). **p < .01 and ***p < .001 Bonferroni corrected post-hoc test. White text on bars in **A** and **B** represent sample sizes (*n*/*N*). (**C**) Mean *I*_Ca_ density-voltage relationships recorded in the presence of H-89 from intact 3-month WT (black circles) and Cav-3OE myocytes (gray squares). Two-way repeated measures analysis of variance (RM ANOVA): voltage *p* < .001, genotype *p* < .05, interaction *p* < .001. (**D**) Corresponding data for 24-month myocytes. Two-way RM ANOVA: voltage *p* < .001, genotype *ns*, interaction *p* < .01. (**E** and **F**) Data presented in **C** and **D**, rearranged to compare within (**E**) WT (two-way RM ANOVA: voltage *p* < .001, age *p* < .001, interaction *p* < .001), or (**F**) Cav-3OE (two-way RM ANOVA: voltage *p* < .001, age *ns*, interaction *ns*) mice. (**G, H**) Effect of PKA inhibition on mean *I*_Ca_ density at 0 mV in the absence (−, from Figure 2) and presence (+) of H-89 measured in intact or DT cells from 3-month and 24-month WT (G) and Cav-3OE (H) mice. Sample sizes for intact cells/hearts in control solution and in the presence of H-89 are provided in Figures 2 and 3, respectively. For DT myocytes, *n/N* were: WT 3 month = 8/3; 24 months = 6/3; Cav-3OE 3 months = 7/2; 24 months = 7/2). *p < .05, **p < .01, and ***p < .001, Bonferroni corrected post-hoc test. (**I**) Calculated *I*_Ca_ density at 0 mV for the whole cell, surface, or t-tubular membranes in WT myocytes in the presence of H-89. (**J**) Corresponding data for Cav-3OE myocytes. *p < .05, **p < .01, and ***p < .001, Student’s *t* test.

To clarify the effect of Cav-3 over-expression at 3 months, particularly whether the reduction in *I*_Ca_ was Cav-3-dependent rather than a result of transgenic modification, Cav-3 scaffolding domain peptide (C3SD peptide) was used (25,32). C3SD peptide interferes with the interaction of Cav-3 with its binding partners, thus reducing the effect of Cav-3 over-expression. Figure 3 shows the effect of C3SD on *I*_Ca_ density measured at 0 mV in 3 months (panel A) and 24 months (panel B), WT and Cav-3OE myocytes. While application of C3SD decreased *I*_Ca_ density in 3 months WT myocytes, as shown previously in rat (25), it *increased I*_Ca_ density in 3-month Cav-3OE myocytes. In contrast, C3SD had no effect on *I*_Ca_ density in 24-month WT or OE myocytes (Figure 3B). These data show that the reduction in *I*_Ca_ in 3-month Cav-3OE myocytes was reversed with C3SD, indicating that the effect of the peptide was independent of Cav-3 expression level, and that Cav-3 dependent regulation of *I*_Ca_ decreased with age.

Age was associated with reduced t-tubular *I*_Ca_ density in WT myocytes (Figure 2C). A possible mechanism for this observation is an age-dependent reduction in constitutive PKA-induced stimulation of t-tubular LTCCs, which is also regulated by Cav-3 (25). We therefore used the PKA-inhibitor H-89 to investigate the role of PKA in the response to age. The mean current density-voltage relationships for *I*_Ca_ recorded in the presence of 20 μmol/L H-89 from 3- and 24-month WT and Cav-3OE intact myocytes are shown in Figure 3C and D arranged allow comparison of the effect of genotype in 3-month (Figure 3C) and 24-month (Figure 3D) myocytes. The same data are shown rearranged in Figure 3E and F to allow comparison of the effects of age in WT (Figure 3E) and Cav-3OE (Figure 3F) myocytes. H-89 decreased *I*_Ca_ density in all groups of cells, regardless of age, presence of t-tubules or genotype (Figure 3G, H), demonstrating constitutive LTCC phosphorylation in both the cell surface and t-tubular membranes, in both young and aged myocytes regardless of Cav-3 over-expression (Figure 3I, J).

More importantly, in the presence of H-89, Cav-3OE persisted in decreasing *I*_Ca_ in intact 3 month, but not in 24-month myocytes, while aging decreased *I*_Ca_ in WT, but not in Cav-3OE, myocytes (Figure 3C–F). Figures 3I and J also show that *I*_Ca_ density was not significantly different at the surface membrane in the four groups of myocytes, suggesting that the observed changes in *I*_Ca_ occurred in the t-tubules (*cf.*Figure 3G, H). These changes were similar to those observed in the absence of H-89 (Figure 2), which suggests that the effects of Cav-3OE on *I*_Ca_ were not due solely to differences in PKA-dependent phosphorylation.

### Effect of Aging and Cav-3OE on Protein Expression

**Figure 4.**
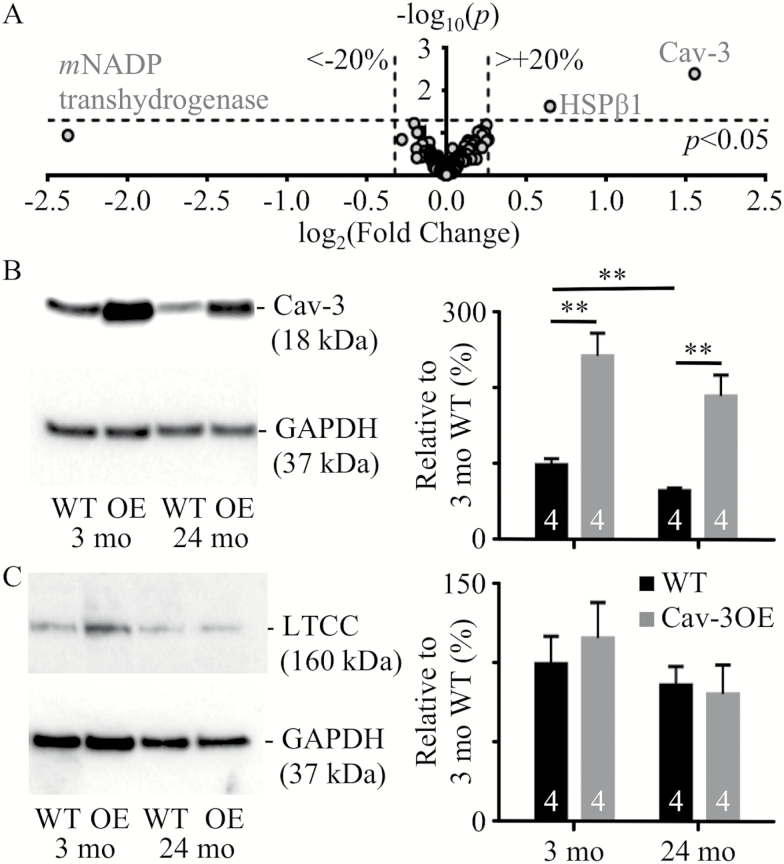
Changes in protein expression with age and Cav-3 over-expression. (**A**) Proteomic analysis of cell lysates from 3 months wild-type (WT) and Cav-3OE mice. Proteins with expression altered in excess of ±20% (marked by vertical dashed lines) are labeled: Cav-3, Heat Shock Protein β1 (HSPβ1) and mitochondrial (*m*) NADP transhydrogenase. Horizontal dashed line indicates limit of statistical confidence (*p* < .05). (**B**) Representative Western blots and mean data for Cav-3 protein expression. (**C**) Representative Western blots and mean data for LTCC protein expression. Mean densities are expressed normalized to glyceraldehyde 3-phosphate dehydrogenase (GAPDH) and 3-month WT and sample sizes are shown within the bars. ** indicates *p* < .01, Student’s *t* test.

To investigate whether compensatory protein changes in the transgenic mice might account for these effects, we performed a proteomic analysis of myocytes from WT and OE mice (Figure 4A). These data showed altered expression in only two proteins: Cav-3 and Heat Shock Protein β1 (HSPβ1) increased by 2.9-fold (*p* < .01) and by 1.6-fold (*p* < .05), respectively. The mechanism underlying the increased expression of HSPβ1 is unclear. Expression of Cav-3 and LTCC was examined by western blotting in 3- and 24-month WT and OE myocytes (Figure 4B, C). Cav-3 expression was ~2.5-fold greater in OE myocytes compared with WT cells at both ages, and aging was associated with 35% and 22% decreases in Cav-3 expression in WT and Cav3-OE myocytes, respectively. Despite the age-related decrease in Cav-3 expression in Cav-3OE myocytes, the level of expression of Cav-3 in 24-month Cav-3OE cells was greater than that in 3-month WT cells (*p* < .05). However, Cav-3OE did not alter the expression of the LTCC α_1c_-subunit at either age. Although mean LTCC expression appeared to decrease by ~15% with age in both groups, this was not statistically significant.

**Figure 5.**
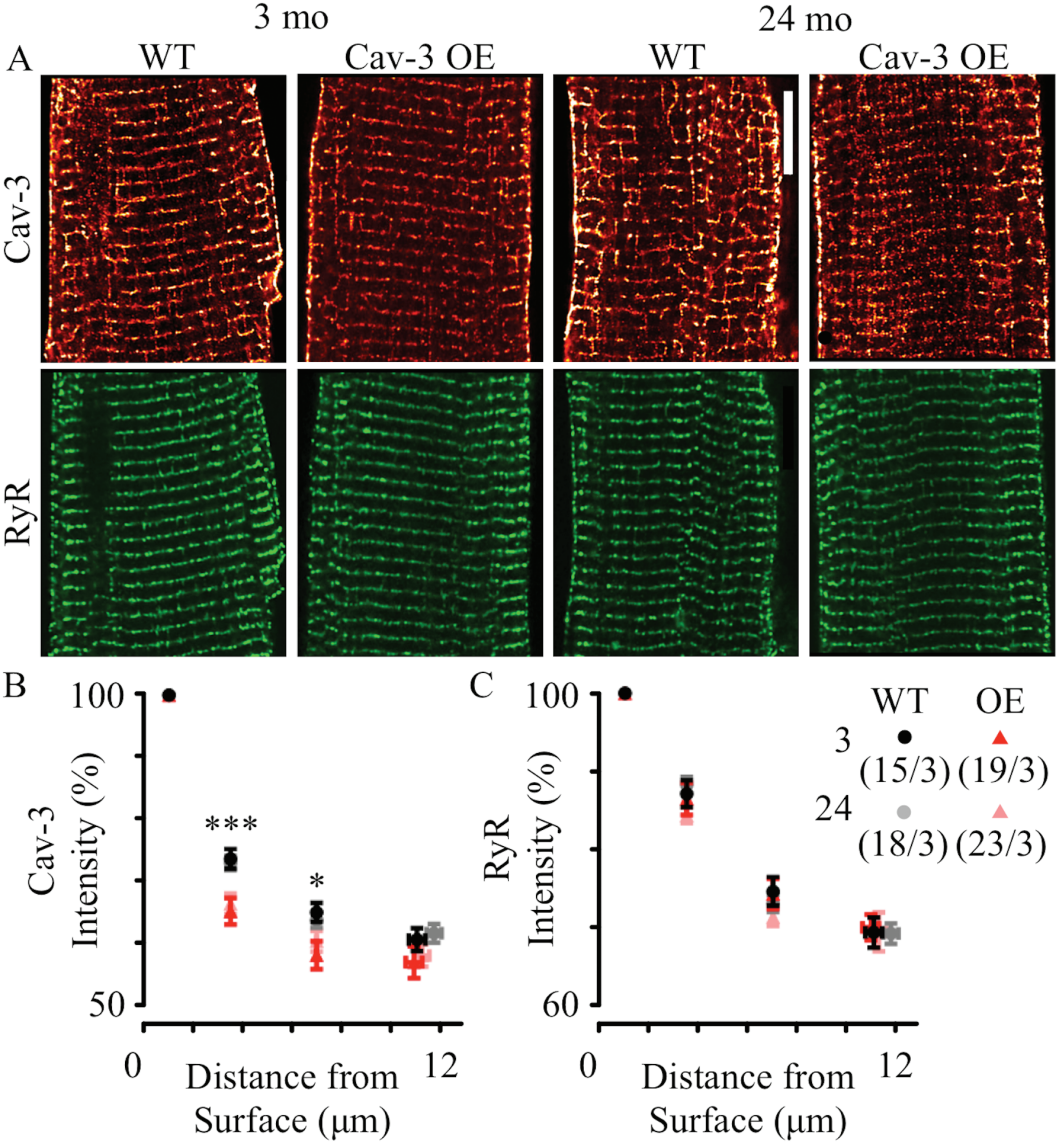
Cav-3 and RyR protein localization in cardiac myocytes with age and Cav-3OE. (**A**) Representative images of Cav-3 (red) and RyR (green) labeling in 3- or 24-month-old cells from wild-type (WT) or Cav-3OE mice. Scale bar indicates 10 μm. The graphs show sarcolemmal (including t-tubules) Cav-3 (**B**) or RyR (**C**) normalized staining intensity as a function of distance from the surface sarcolemma. *p < .05 and ***p < .001 by two-way analysis of variance for differences between WT and Cav-3OE.

The effect of Cav-3 over-expression on Cav-3 protein localization was examined by immunocytochemistry in WT and OE myocytes of both ages (Figure 5A). Cav-3 staining was observed at the surface of the cell and in regular, transverse striations with a periodicity of ~1.8 μm, near RyR staining (lower panels), which supports the idea that the majority of Cav-3 antigenicity is at the sarcolemmal membranes (including t-tubules). The intensity of the sarcolemmal Cav-3 labeling decreased from the cell surface to the interior in all groups (Figure 5B). This gradient was more pronounced in Cav-3OE myocytes at both ages than in the corresponding WT myocytes, suggesting a modest (~10%) decrease in the relative amount of Cav-3 staining at the t-tubules compared to the surface. There were no changes in RyR labeling due to age or genotype (Figure 5C). Using RyR labeling as a marker of the z-disc (since its distribution was not altered between groups), colocalization of Cav-3 with RyR labeling tended to reduce with Cav-3 over-expression: from 68 ± 2% (*n*/*N* = 15/3) to 58 ± 3% (*n*/*N* = 19/3) in 3-month cells and 62 ± 2% (*n/N* = 18/3) to 59 ± 2% (*n/N* = 23/3) in 24-month cells (data not shown, *p* < .05, two-way ANOVA). These data suggest that Cav-3OE may be associated with a mildly altered Cav-3 localization (particularly in 3-month myocytes).

### SR Ca Release, the Systolic Ca Transient and SR Ca Content

Whole-cell Ca transients recorded from field-stimulated (0.1, 0.2, and 1.0 Hz) myocytes showed only modest changes in Ca transient amplitude and time course with age and Cav-3 over-expression (Supplementary Figures S2A and S2B). There were also no marked differences in time-to-peak or time-to-half decay between groups: all demonstrated the characteristic reduction in duration with increasing stimulation frequency, although the Cav-3OE groups show slightly longer durations (Supplementary Table 3).

Closer examination of Ca release near t-tubules using simultaneous measurement of membrane potential and intracellular Ca also revealed little difference between groups (Supplementary Figure S2C). The upper panels of Supplementary Figure S2C shows the rising phase of Ca transients scanned along the line of a t-tubule in representative myocytes from 3- (left panels) and 24-month (right panels) WT and Cav-3OE myocytes. Lower panels show the time of AP upstroke (yellow), initiation of Ca release (red), and maximum rate of rise of Ca (green). Latency to the initiation (Supplementary Figure S2D) or maximum rate (Supplementary Figure S2E) of Ca release were not altered by age, nor by Cav-3OE. The heterogeneity of Ca release (the dispersion, or standard deviation of Ca release latencies) was also unaltered (Supplementary Figure S2F). The amplitude of the Ca release induced by rapid application of caffeine (10 mM), an index of SR Ca content, was not significantly different between 3 and 24 months in WT (∆F/F_0_ = 3.6 ± 0.2, *n/N* = 16/3 vs 3.4 ± 0.3, *n/N* = 20/3) or Cav-3OE myocytes (∆F/F_0_ = 3.2 ± 0.3, *n/N* = 15/3 vs 4.1 ± 0.4, *n/N* = 9/3), or between genotypes. Thus, it appears that the observed changes in t-tubular *I*_Ca_ are accompanied by only modest changes in Ca handling.

## Discussion

The present study shows, for the first time, that the reduction in *I*_Ca_ density of male ventricular myocytes with age occurs predominantly at the t-tubules. The study is also the first to investigate the involvement of t-tubule structure and function, and the role of Cav-3, in aging. In addition to the decreased t-tubular *I*_Ca_ density with age, the major findings of the present study were that: (i) although Cav-3OE augmented Cav-3 expression in both age groups, it did not prevent the reduction of Cav-3 expression with age; (ii) despite large decreases in Cav-3 expression with age, changes in t-tubule organization and Cav-3 localization were modest; (iii) age-dependent cellular hypertrophy was not ameliorated by transgenic overexpression of Cav-3; (iv) overexpression of Cav-3 reduced t-tubular *I*_Ca_ density at 3-mo, and removed the age-dependent reduction in *I*_Ca_ so that the current was maintained at 24-mo; (v) in contrast to 3 months, at 24 months, *I*_Ca_ did not appear to be Cav-3 dependent, as demonstrated by the lack of effect of Cav-3OE and application of C3SD; and (vi) neither aging nor Cav-3OE appeared to have pronounced effects on Ca release at steady state.

### Cav-3 Expression and Localization

Cav-3 protein expression was reduced with age in WT myocytes (Figure 4B), consistent with previous reports in mice (28,29), although localization of Cav-3 staining at the t-tubules did not change with age. Cav-3OE did not prevent age-dependent loss of Cav-3 although, as might be expected, Cav-3 expression was increased at both ages above that seen in 3-month WT (31). However, Cav-3OE resulted in a steeper drop in Cav-3 staining intensity from surface sarcolemma to t-tubules, suggestive of a partial disruption of Cav-3 protein association with the t-tubules. Alternatively, the steeper drop in staining intensity from surface to interior in Cav-3OE might be a result of the preferential localization of overexpressed Cav-3 to the surface membrane. The degree of colocalization of Cav-3 with RyR labeling appears large compared to that reported by others (36,37). However, in the present study, RyR labeling was used simply as a marker for the z-disc. Due to microscope blurring, the analysis of relatively un-processed confocal microscopy data in the present study would have over-estimated the absolute colocalization. Thus, the present study is not inconsistent with the previous studies of Scriven et al. (2005) and Wong et al. (2013) (36,37). Nevertheless, our simple, but straight-forward, approach enables comparison of Cav-3 protein localization between groups (ie, age and genotype).

### Cell Morphology and t-Tubule Capacitance

Age was associated with cellular hypertrophy and reduction in expression of Cav-3, consistent with previous studies (1,28,29). Loss-of-function mutations and knockout of Cav-3 are also associated with cardiac hypertrophy, consistent with a role for loss of Cav-3 expression in age-related hypertrophic signaling (38,39). However, in this study, the age-dependent hypertrophy was greater in myocytes from Cav-3OE than WT mice, demonstrating that the hypertrophy was increased, and not ameliorated, by overexpression of Cav-3. Nevertheless, the data are consistent with the involvement of Cav-3 in hypertrophic signaling pathways in cardiac myocytes (20–22). Presumably, due to the intimate involvement of Cav-3 in hypertrophic signaling pathways, either loss or gain of Cav-3 function can cause hypertrophy. For example, the hypertrophic cardiomyopathy caused by knockout of Cav-3 was associated with loss of caveolae and increased p42/p44 MAPK signaling (39) whereas the cardiac-specific transgenic overexpression of Cav-3, as used in the present study, results in increased numbers of caveolar signalsomes (31).

Age was also associated with an increase in the fraction of the membrane in the t-tubules in both genotypes determined using cell capacitance (Figure 1), while imaging data revealed only a modest reduction in t-tubule density with age (Figure 1). The apparent discrepancy cannot be explained by differences in DT efficiency (see Methods section). However, while at 3 months, DT cell size was not markedly different from that of intact cells in either WT or Cav-3OE cells, 24-month DT cells were smaller than their intact counterparts (Figure 1, Supplementary Tables S1 and S2). Cell sizes from DT cells provide a measure of the surface sarcolemmal membrane capacitance. Considering Laplace’s Law, the greater wall stress caused by the formamide-induced osmotic shock in hypertrophied cells at 24 months compared with the smaller 3-month cells may have resulted in increased death of the larger cells and thus selected smaller cells in the 24-month group. Given the larger dimensions of WT and Cav-3OE myocytes at 24 months compared with 3 months, the model predicted a 22% increase in total membrane area with age for WT myocytes and a 55% increase in total membrane area for Cav-3OE cells in the absence of any change in t-tubule density, which agrees well with the observed increase in cell capacitance with age (24% and 55%, Figure 1 and Supplementary Table S2). The corresponding fraction of membrane in the t-tubules for 24-month WT myocytes was 45%, which also agrees well with experimental data (52%, Figure 1 and Supplementary Table S2). A similar analysis for changes in Cav-3OE myocytes with age revealed a 55% increase in total membrane area and a fraction of membrane that is in the t-tubules of 42%, which also agree well with those obtained experimentally (55% and 45%, respectively, Figure 1 and Supplementary Table S2). Taken together, these data suggest little change in t-tubule structure with age or with over-expression of Cav-3. Although Cav-3 has been implicated in the development of t-tubules and the cardiac-specific overexpression of Cav-3 has previously been reported to increase numbers of caveolae in heart muscle (19,31,40), it is striking that in the present study overexpression of Cav-3 had no effect on t-tubule morphology. Presumably other structural proteins, such as BIN-1, are also required for t-tubule development (41).

Over-estimation of t-tubule capacitance due to small DT 24-month myocytes might lead to under-estimation of t-tubular *I*_Ca_ density. However, applying the model described above to correct t-tubular *I*_Ca_ and capacitance results in little change in the data: in 3-month cells, the calculated t-tubular *I*_Ca_ density is unchanged (compare Supplementary Table S1 to Figure 2C), and in 24-month cells, the corrected t-tubular *I*_Ca_ density for WT and OE myocytes is −8.2 ± 0.9 and −10.1 ± 1.2 pA/pF, respectively (Supplementary Table S1). Thus, the interpretation of the data is unchanged: age significantly decreases t-tubular *I*_Ca_ density in WT (−41%, *p* < .01), but not in OE (*ns*, *t* test). Such consideration of the effect of cell size on t-tubular membrane fraction and current densities may be important in any investigation of interventions that cause changes of cell size and has not, to our knowledge, been considered previously.

### Distribution and Regulation of I_Ca_

The present study shows that the age-dependent decrease in *I*_Ca_ density occurs primarily at the t-tubules (Figure 2) and is associated with a decrease in Cav-3 expression (Figure 4). Cav-3 has previously been shown to associate with LTCC and elements of the *β*_2_-adrenergic/cAMP-dependent pathway at the t-tubule and mediate PKA-dependent constitutive stimulation of t-tubular *I*_Ca_ (25,27,42). Pretreatment of cells with C3SD peptide reduced *I*_Ca_ density in 3-month WT, but not in 24-month WT cells (Figure 3), suggesting that the reduction in *I*_Ca_ density with age is associated with the loss of a Cav-3-dependent mechanism that augments t-tubular *I*_Ca_ density. However, the decrease of *I*_Ca_ density was *not* a consequence of reduced constitutive PKA-dependent stimulation of *I*_Ca_ with age, because application of H-89 caused a robust decrease in whole-cell and t-tubular *I*_Ca_ density in both 3-month (−62% and −58%, respectively) and 24-month (−64% and −59%, respectively) cells. Nor was the age-dependent decrease in *I*_Ca_ density a consequence of reduced LTCC expression (Figure 4). Age has been associated with redistribution of Cav-3 from cholesterol-rich to cholesterol-free membranes in heart muscle, indicating a loss of caveolin from caveolae with age (43). This redistribution may underlie the apparent loss of association of Cav-3 with LTCC and the cAMP signaling pathway in the t-tubule membrane in 24-month myocytes so that the constitutive regulation of *I*_Ca_ became insensitive to C3SD but retained sensitivity to PKA inhibition. However, localization of Cav-3 to the t-tubules was not reduced with age (Figure 5). Moreover, the age-dependent reduction in *I*_Ca_ density was associated with an increased total membrane area and an increased fraction of membrane in the t-tubules (Supplementary Table S2 and Supplementary Figure S1).

Over-expression of Cav-3 was associated with reduced t-tubular *I*_Ca_ density in 3-month myocytes, suggesting that *I*_Ca_ is decreased by either inhibition or overexpression of Cav-3, and consistent with a role for Cav-3 in determining basal t-tubule *I*_Ca_ density in myocytes from young animals (25). The reduction of *I*_Ca_ caused by OE was not due to a decrease in LTCC expression (Figure 4), but might reflect slightly less constitutive PKA stimulation of *I*_Ca_ since application of H-89 to 3-month Cav-3OE cells was associated with a smaller reduction in whole-cell (−56%) and t-tubular (−47%) *I*_Ca_ compared to WT (see Figure 3G, H). A possible explanation is that Cav-3OE resulted in mis-location of a fraction of the protein that, in consequence, was unable to perform its native task(s) but competed for binding partners with normally-located Cav-3. This idea is consistent with the observation that application of C3SD peptide *increases I*_Ca_ density in 3-month Cav-3OE myocytes (Figure 3). Imaging data also showed that there may be some relocation of Cav-3 with over-expression, as Cav-3OE cells showed reduced relative Cav-3 staining intensity at the t-tubules (Figure 5).

Interestingly, in contrast to the decrease observed in WT myocytes, there was no change in whole cell or t-tubular *I*_Ca_ density during aging in Cav-3OE myocytes (Figure 2). The preservation of *I*_Ca_ density at the t-tubules in 24-month Cav-3OE myocytes occurs despite a 77% increase in t-tubular surface area due to age-dependent hypertrophy. This suggests maintenance of t-tubular *I*_Ca_ as a result of Cav-3 overexpression. Despite the reduction in Cav-3 expression with age in Cav-3OE myocytes, expression of the protein remained approximately twofold greater than in 3-month WT myocytes (Figure 4). Thus, the data are consistent with the proposal that overexpression of Cav-3 protected against the age-dependent loss of LTCC function from the t-tubules. Nevertheless, Cav-3 staining intensity at the t-tubules appeared reduced compared to 24-month WT myocytes (Figure 5), suggesting disruption of Cav-3 localization in 24-month Cav-3OE cells. The mechanism by which Cav-3 over-expression protected against the age-dependent loss of t-tubular *I*_Ca_ remains unclear. H-89 caused a similar decrease in *I*_Ca_ density in 24-month WT and OE myocytes (Figure 3), suggesting that the reduced Cav-3 expression with age in Cav-3OE was sufficient to alleviate the small inhibitory effect of overexpression on the Cav-3/PKA-dependent pathway that was evident at 3 months. This is consistent with the results obtained in the presence of C3SD, which showed no effect on *I*_Ca_ in 24-month Cav-3OE cells (Figure 3). In any case, while H-89 reduced whole-cell and t-tubule *I*_Ca_ density in both 3-month and 24-month Cav-3OE cells, there was no difference in current densities between the two ages, indicating that the protective effect of Cav-3 over-expression against age-dependent loss of t-tubular *I*_Ca_ was independent of constitutive regulation by PKA.

### Excitation-Contraction Coupling

The ~50% decrease in t-tubular *I*_Ca_ density with age was not associated with altered Ca release at the t-tubule in the present study: latency between action potential upstroke and Ca release, heterogeneity of Ca release along the t-tubule and Ca transient properties were not significantly affected by age (Supplementary Figure S2 and Supplementary Table S3). This is consistent with previous studies that have shown no age-dependent differences in Ca transient amplitude or duration when cells were stimulated at frequencies similar to those used in the present study, although at higher frequencies the Ca transient may be smaller and slower with age (9,44). There were no changes in RyR distribution (Figure 5), LTCC expression (Figure 4), or SR Ca content. Thus, the unaltered Ca release may be explained by: (i) the highly nonlinear relationship between *I*_Ca_ and SR Ca release (45,46) so that there is effectively a functional reserve in *I*_Ca_; (ii) since a significant proportion of LTCCs are located outside the dyad, reduction in *I*_Ca_ density in this population would have little effect on CICR. The former is supported by the observation that absolute t-tubular *I*_Ca_ is unaltered while fractional t-tubule area increased in myocytes from old animals (Supplementary Table S2), in contrast to the decrease in absolute t-tubular *I*_Ca_ with no change in t-tubular membrane area observed in heart failure (26). The latter is supported by recent evidence of a role for Cav-3 in the regulation of nondyadic LTCC in cardiac muscle (47,48).

## Supplementary Material

Supplementary data is available at *The Journals of Gerontology, Series A: Biological Sciences and Medical Sciences* online.

Supplementary Figs 1

Supplementary Figs2

Supplementary Material

## Funding

This work was supported by the British Heart Foundation (BHF RG/12/10/29802 [C.H.O., A.F.J., and M.B.C.], PG/14/65/31055 [C.H.O., A.F.J.]) and grants from the National Institutes of Health (NIH HL091071 [H.H.P.], HL107200 [H.H.P. and D.M.R.], HL066941 [H.H.P. and D.M.R.], HL115933 [H.H.P. and D.M.R.], AG052722 [H.H.P.]) and the Veterans Affairs Administration (VA Merit BX001963 [H.H.P.] and BX000783 [D.M.R.]).

## Conflict of Interest

None reported.
