# Supplementary figures and images for: The Effects of Aging on the Regulation of T-Tubular ICa by Caveolin in Mouse Ventricular Myocytes
Source: J Gerontol A Biol Sci Med Sci. 2017 Dec 9;73(6):711–9. doi: 10.1093/gerona/glx242 (PMC5946816; doi:10.1093/gerona/glx242)

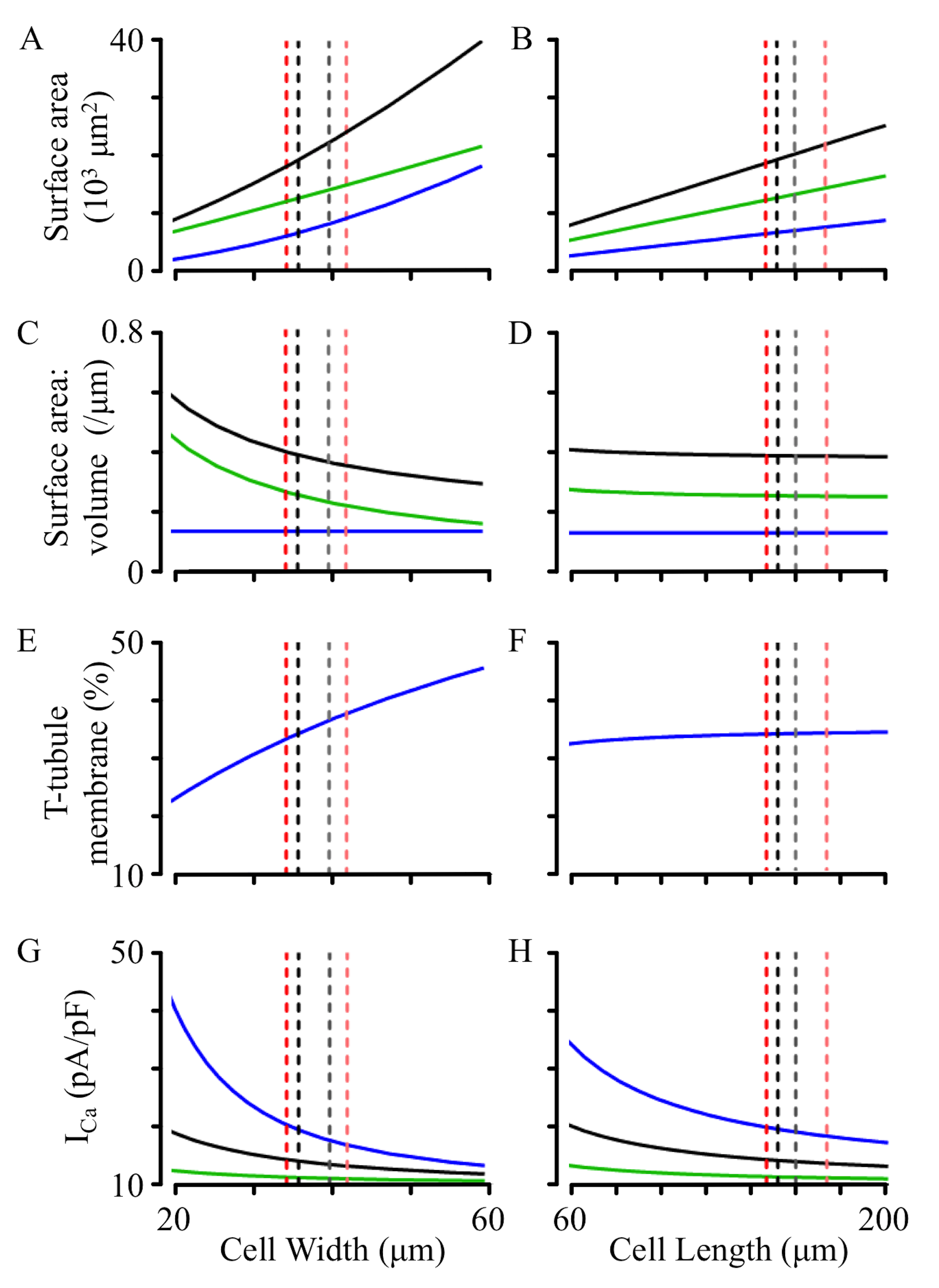

Supplement: Supplementary Figs 1 [file glx242_suppl_supplementary_figs1.png]

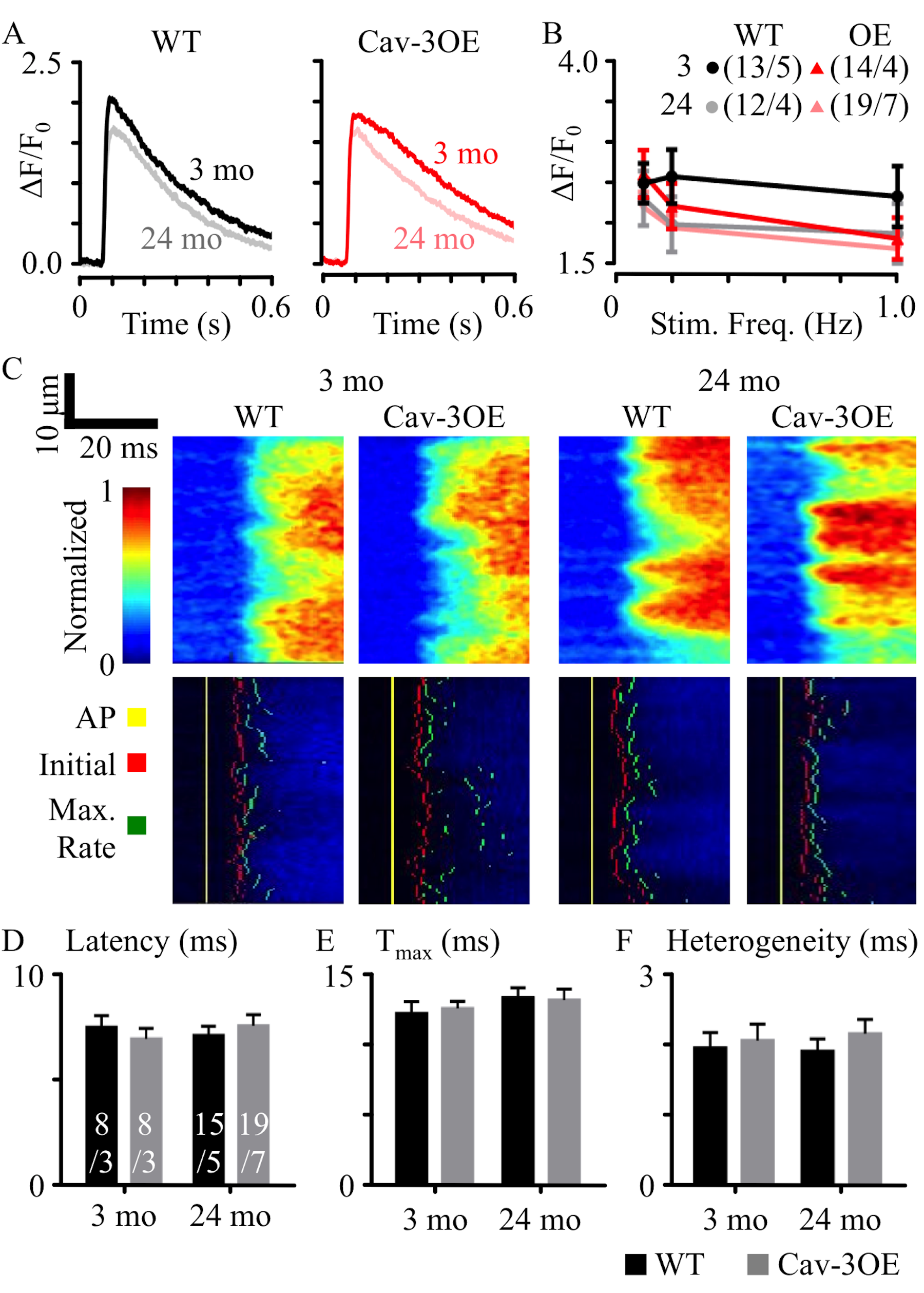

Supplement: Supplementary Figs2 [file glx242_suppl_supplementary_figs2.png]
