# Supplementary material for: The Effects of Aging on the Regulation of T-Tubular ICa by Caveolin in Mouse Ventricular Myocytes
Source: J Gerontol A Biol Sci Med Sci. 2017 Dec 9;73(6):711–9. doi: 10.1093/gerona/glx242 (PMC5946816; doi:10.1093/gerona/glx242)
Supplement: Supplementary Material [file glx242_suppl_supplementary-material_final.docx]

**Supplementary Material**

**Methods**

***Myocyte detubulation***

Myocyte detubulation (DT) – the physical and functional uncoupling of the t-tubules from the surface membrane – was achieved using formamide-induced osmotic shock (1). Briefly, cells were exposed to 1.5 M formamide in standard perfusion solution (see below) for 3 min and then rapidly resuspended in formamide-free solution. Analysis of membrane-stained intact and DT myocytes (see below) showed that DT efficiency, the percentage of tubules removed, was 87 ± 5 % (*n/N*, Intact=22/4, DT=15/2) and 89 ± 4 % (*n*/*N*, Intact=30/4, DT=18/2) in 3-mo WT and OE myocytes, respectively. This was unchanged in 24-mo WT and OE myocytes, which showed DT efficiencies of 92 ± 7 % (*n*/*N*, Intact=15/3, DT=10/2) and 90 ± 6 % (*n*/*N*, Intact=34/7, DT=16/2), respectively.

**Confocal imaging and analysis of cell membrane and protein distribution**

Cell surface membrane was labelled by incubating cells with 5 μM di-8-ANEPPS for 15 min. Cav-3 and RyR were labelled using standard immunocytochemical techniques; cells were fixed in 2% paraformaldehyde in phosphate-buffered saline (PBS) for 10 min, permeabilized with 0.1% Triton-X100 for 10 min and incubated in 10% goat serum for 1 hr, at room temperature. Cells were then labelled with rabbit α-Cav-3 antibody (1:250 Ab2912; Abcam, Cambridge, UK) and mouse α-RyR antibody (1:100 MA3-913; Thermo Fisher) in PBS containing 2% goat serum and 2% bovine serum albumin at 4°C overnight, then with Alexa 488 goat anti-rabbit antibody (1:200; A11037, Thermo Fisher) and Alexa 594 goat anti-mouse antibody (1:400, A11005, Thermo Fisher) before being mounted in Mowiol 4-88 containing 2.5% 1,4-diazabycyclo-[2,2,2]-octane (Sigma-Aldrich, MO, USA) on standard glass slides and stored overnight to cure.

Cells stained with di-8-ANEPPS were imaged using a FluoView 1200 confocal system (Olympus, Japan), with a silicone immersion objective with 1.25 numerical aperture (NA). Excitation/emission was 488/500-600 nm, and voxel size was ~90 nm in-plane (*x-y*) and 250 nm along the optical axis (*z*). Protein labeling was imaged using an LSM 880 confocal system (Zeiss, Germany) with the Airyscan detector in super-resolution mode and a 1.4 NA oil immersion objective. RyR and Cav-3 signals were acquired sequentially using excitation/emission at 594/>605 and 488/495-550 nm, respectively. Voxel size was ~40 nm in *x-y* and 180 nm in *z*.

Image stacks were analyzed using custom routines written in MATLAB R2014b (Mathworks, Inc., MA, USA). For t-tubule staining, image stacks were deconvolved using the Richardson-Lucy algorithm and the point-spread function (PSF) measured from sub-resolution fluorescent microspheres. To assess the density and organization of the t-tubules, staining was skeletonized using the Sobel operator, which calculates local derivatives that can be used to extract the lengths of longitudinal and transverse tubules (e.g. see reference (2)). The sum of both yields total tubule length and all were normalized to cell area. Sarcolemmal staining intensity was measured by a global threshold determined by an Otsu method, as given by *graythresh()* in Matlab and verified by eye, where brightly stained pixels in the Cav-3 signal were considered to be in the surface sarcolemma and t-tubular membranes. Protein distribution was assessed by examining labeling intensity as a function of distance from the cell surface. Intensity was measured within bins of 0-2, 2-5, 5-9 μm from the surface normalized to the fluorescence measured in the surface bin. Co-localization was quantified using Mander’s Coefficients and were applied to un-processed data.

**Recording and analysis of I_Ca_**

Myocytes were placed in a chamber mounted on a Nikon Diaphot inverted microscope. Membrane currents and cell capacitance were recorded using the whole-cell patch-clamp technique, using an Axopatch 200B (Molecular Devices, CA, USA) and Digidata 1322A A/D converter (Axon Instruments). pClamp 10 software (Molecular Devices) was used for data acquisition and analysis. Patch pipette resistance was typically 1.5-3 MΩ when filled with pipette solution. Pipette capacitance and series resistance were compensated by >70%. *I*_Ca_ was elicited from a holding potential of -80 mV: after a 100 ms step depolarization to -40 mV (to inactivate *I*_Na_), step depolarizations were applied every 5 s to voltages ranging between -50 and +80 mV (in 10 mV steps) for 500 ms before repolarization to the holding potential. *I*_Ca_ amplitude was measured as the difference between the peak inward current and current at the end of the depolarizing pulse, normalized to cell capacitance and reported as current density (pA/pF). Similarly, surface membrane current density was obtained from currents measured in DT myocytes. The membrane current density in the t-tubules was calculated by subtracting surface from whole-cell currents and dividing by the difference in capacitance between intact and DT cells, with correction for incomplete DT (see above) as described previously (3).

**Proteomic analysis**

Analysis of proteins from samples of isolated myocytes (3 hearts for both WT and OE) was performed by the University of Bristol Proteomics Facility using tandem mass tags (TMTs) (Thermo Fisher Scientific, UK) as described previously (4). Briefly, aliquots of each sample (100 μg) were subjected to tryptic digestion and resultant peptides labelled with TMT sixplex reagents according to the manufacturer’s protocol. The combined samples were fractionated using a Dionex Ultimate 3000 nano high-performance liquid chromatography system in line with an LTQ-Orbitrap Velos mass spectrometer (Thermo Scientific, UK). The Orbitrap was set to analyze the survey scans at 60,000 resolution (at m/z 400) in the mass range m/z 300 to 1800 and the top ten multiply charged ions in each duty cycle selected for MS/MS fragmentation. The raw data files were processed and quantified using Proteome Discoverer software v1.2 (Thermo Scientific, UK) with the reverse database search option enabled and all peptide data filtered to satisfy a false discovery rate (FDR) of 5%. These were then searched against the Uni-Prot mouse database (16,747 entries) using the SEQUEST (Ver. 28 Rev. 13) algorithm to assign protein identity.

**Western Blot analysis**

10 µg samples of isolated myocyte lysates were run on 4-15% gradient SDS-PAGE gels and transferred onto Immobilon-P membrane. The blot was probed with α-Cav-3 antibody (BD Transduction Laboratories; 610420), α-LTCC (Alomone; ACC-003) or anti-GAPDH (Sigma; G9545) and protein bands visualized using relevant peroxidase conjugated secondary antibodies with chemilumenescence and a G:BOX Chemi XT4 imaging system (SynGene). Band density was quantified using Image J (<http://imagej.nih.gov/ij/>) and normalized to GAPDH.

**Confocal measurement of intracellular Ca and/or membrane potential**

To monitor intracellular Ca, myocytes were incubated in 5 μM Fluo-4/AM (Thermo Fisher Scientific, MA, USA) for 25 min. To monitor membrane potential simultaneously, in a subset of experiments, di-4-AN(F)EPPTEA (0.5–1 μg/ml; supplied by Dr Leslie Loew (5)) was added for the last 15 min of incubation. Cells were then washed and stored in perfusion solution. Cells were imaged in a perfusion chamber mounted on a FluoView 1200 (as above). For Ca imaging, line-scans along the length of the cell were recorded during 488 nm excitation and 490-590 nm collection. For simultaneous voltage and Ca imaging (3), line-scans along a single t-tubule were recorded at 0.488 ms/line, during excitation at 514 nm and collection of emissions between 516-560 nm for Ca, and 590-690 nm for voltage. Cells were field-stimulated at 0.1, 0.2 and 1.0 Hz to steady-state at 1.5× threshold using parallel Pt wires, in alternating order between cells.

**Results**

**A geometric model of the relationship between cell size and surface membrane area.**

The surface area of objects generally increases non-linearly with volume. For a cardiac myocyte, this relationship is unclear, primarily due to the t-tubular network, which greatly increases cell membrane area for a given cell volume. It is, however, important, given the changes in cell size and t-tubule morphology reported in many physiological and pathological conditions, and their implications for cell function. To investigate how cell size might affect membrane area, a geometric model was constructed.

The surface sarcolemma (SS) of a myocyte was approximated by a closed elliptical cylinder (e.g. (6)). The cell volume, *V*, is:

$$V=\pi\cdot r_{x}\cdot r_{y}\cdot l$$

Eqn. 1

where r_x_, r_y_ and l are cell half-width, half-depth and cell length, respectively. The SS area, SA_SS_, is given by Eqn. 2, where *r_z_* and *P* are cell half-depth and circumference (or perimeter) of the ellipse, respectively. Cell depth was estimated as one third of measured cell width (6) and *P* was calculated using the Ramanujan approximation as given (7).

Eqn 2:

$${SA}_{SS}=2\pi\cdot r_{x}\cdot r_{z}+P\cdot l$$

where,

$$P\approx\pi\cdot(r_{x}+r_{z})\cdot\left( 1+\frac{3h}{10+\sqrt{4-3h}} \right)$$

and,

$$h=\frac{{(r_{x}-r_{z})}^{2}}{{(r_{x}+r_{z})}^{2}}$$

Thus, SA_SS_ is equivalent to the total membrane area of an ideal detubulated (DT) myocyte (1).

T-tubules were approximated as circular cylinders that invaginated the surface sarcolemma, penetrating the cell volume. The radius of the tubules, *r_TT_*, was set to 0.12 μm (8,9) and the length of t-tubule (*l_TT_*) per cell volume (i.e. t-tubule density, *l_TT_/V*) was set to 0.17 μm/μm^3^ to give a percentage of membrane that is t-tubular (%_TT_, see below) of 34%, as observed in 3 mo old WT myocytes (Table 1). The surface area of t-tubules, SA_TT_, is therefore, given by Eqn. 3 and %_TT_ given by Eqn. 4.

$${SA}_{TT}=l_{TT}\cdot2\pi\cdot r_{TT}$$

Eqn. 3

$${\%}_{TT}=\frac{{SA}_{TT}}{{SA}_{Total}}$$

where,

$${SA}_{Total}={SA}_{SS}+{SA}_{TT}$$

Eqn. 4

To test the effect of cell width on membrane area, cell length was set to 151.5 μm (the mean length in 3 mo WT myocytes; Figure 1) and cell width varied. The relationship between SS, TT and total SA and cell width is shown in Fig. S1A. SA_Total_ (black line) increases non-linearly with cell width. This non-linearity is primarily due to t-tubules, as shown by comparing the relationships between SA_TT_ (blue line) and SA_SS_ (green line) vs. cell width. As would be expected, an increase in cell length increases these SAs linearly, due to repetition of the sarcomere. This is shown in Fig. S1B, where cell width was set to 35.5 μm (the mean width in 3 mo WT myocytes; Figure 1) and cell length varied.

The surface area to volume ratio (SA/V) of the cell decreases non-linearly with cell width and length (Fig. S1C and D), although the effect of cell length is relatively small. Fig. S1C and D also show that the changes in SA/V occur only at the SS, since t-tubule density (*l*_TT_/V), and therefore, SA_TT_/V, is held constant in these calculations, although the percentage of membrane that is in the t-tubules (Fig. S1E and F) increases non-linearly with cell width and length. The effect of these changes on SS and TT *I*_Ca_ density, assuming no change in absolute *I*_Ca_ at either site, is shown in Fig. S1G and H.

The curves in Fig. S1A and B illustrate the problem that can arise due to reduced DT cell size, which causes SS capacitance to be under-estimated and consequently, TT capacitance to be over-estimated. The magnitude of this effect was estimated using this model by calculating the SA_Total_ of an average cell (e.g. WT, 3 mo, Table S1), then subtracting the SA_SS_ as calculated from its associated DT cell (e.g. 3 mo WT DT, Table S1) to yield the apparent SA_TT_ and %_TT_. This was repeated for the aged counterpart (e.g. WT 24 mo) to estimate the apparent percentage increase in SA_TT_ and %_TT_ with age. Note that since the percentage increases are used, these results are relatively insensitive to parameters such as tubule diameter.

**Figure S1.**  The effect of cell width (left panels) and length (right panels) on t-tubule (TT, blue line), surface sarcolemma (SS, green line) and total (black line) membranes. (A & B) show absolute membrane surface area; (C & D) show ratio of membrane surface area to cell volume; (E & F) show the percentage of membrane that is t-tubule (%_TT_). (G & H) show *I*_Ca_ density (pA/pF) if t-tubular and surface membrane *I*_Ca_ (pA) are kept constant. Cell length was 151.5 μm for (A, C & E) and cell width was 35.6 μm for (B, D & F), which were the mean cell dimensions in cells from 3 mo WT animals. These dimensions are indicated by the black (3 mo WT), the gray (24 mo WT), red (3 mo Cav-3OE) and pink (24 mo Cav-3OE) dashed lines.

**Figure S2**. Effect of age and Cav-3OE related changes on E-C coupling. (A) Representative Ca transients stimulated at 0.2 Hz from 3- or 24-mo cells isolated from WT or Cav-3OE mice. (B) Mean Ca transient amplitude at 0.1, 0.2 and 1.0 Hz. (C) Representative line-scan images and corresponding latency analysis of Ca transients evoked at 0.1 Hz. AP (yellow), ‘initial’ (red) and ‘maximum’ (green) indicate the time of AP upstroke, the initiation of Ca release and Ca transient maximum rate of rise, respectively. (D) Mean latency (corresponds to time from AP to ‘initial’ in panel A). (E) Mean time to maximum rate of rise (time from AP to ‘maximum’ in panel A). (F) Mean release heterogeneity (standard deviation of latency along the t-tubule).

**References**

1. Kawai M, Hussain M, Orchard CH. Excitation-contraction coupling in rat ventricular myocytes after formamide-induced detubulation. Am J Physiol. 1999;**277**:H603-H609.

2. Crossman DJ, Young AA, Ruygrok PN, Nason GP, Baddelely D, Soeller C*, et al.* t-tubule disease: Relationship between t-tubule organization and regional contractile performance in human dilated cardiomyopathy. J Mol Cell Cardiol. 2015;**84**:170-178.

3. Bryant SM, Kong CHT, Watson J, Cannell MB, James AF, Orchard CH. Altered distribution of I_Ca_ impairs Ca release at the t-tubules of ventricular myocytes from failing hearts. J Mol Cell Cardiol. 2015;**86**:23-31.

4. Littlejohns B, Heesom K, Angelini GD, Suleiman M-S. The effect of disease on human cardiac protein expression profiles in paired samples from right and left ventricles. Clin Proteom. 2014;**11**:34.

5. Yan P, Acker CD, Zhou W-L, Lee P, Bollensdorff C, Negrean A*, et al.* Palette of fluorinated voltage-sensitive hemicyanine dyes. Proc Natl Acad Sci USA. 2012;**109**:20443-20448.

6. Boyett M, Frampton J, Kirby M. The length, width and volume of isolated rat and ferret ventricular myocytes during twitch contractions and changes in osmotic strength. Exp Physiol. 1991;**76**:259-270.

7. Ramanujan S. Modular equations and approximations to π. . Quart J Math. 1914;**XLV**:350-372.

8. Bossen EH, Sommer JR, Waugh RA. Comparative stereology of mouse atria. Tissue & Cell 1981;**13**:71-77.

9. Hayashi T, Martone ME, Yu Z, Thor A, Doi M, Holst MJ*, et al.* Three-dimensional electron microscopy reveals new details of membrane systems for Ca^2+^ signaling in the heart. J Cell Sci. 2009;**122**:1005-1013.

**Table S1. Estimation of the effect of reduced DT cell size on calculated t-tubular *I*_Ca_ density.** The expected surface sarcolemmal membrane area from intact cell size measurements and that from DT cell size measurements were calculated using the described geometric model. They were then compared (DT/Intact) to give the extent that *I*_Ca_ might have been under-estimated. For example, since in the WT 24 mo group, DT cells were smaller than Intact cells, the calculated *I*_Ca_ would have been 0.859 of that expected if DT and Intact cells had been the same size. The original *I*_Ca_ densities for Intact, surface sarcolemmal and t-tubular membranes are shown. The estimated *I*_Ca_ densities corrected for smaller DT cell size are given below. Data was subjected to 2-*way* ANOVA. Interaction (I) *= *p*<0.05, **= *p*<0.01, ***= *p*<0.001. For *post hoc* tests: * = *p*<0.05, ** = *p*<0.01, *** = *p*<0.001 vs 3 mo WT cells; $ = *p*<0.05, $$ = *p*<0.01, $$$ = *p*<0.001 vs 3 mo Cav-3OE cells; # = *p*<0.05, ## = *p*<0.01, ### = *p*<0.001 vs 24 mo WT cells. Differences in *I*_Ca_ density at the t-tubular membrane was assessed with Student’s t test where **= *p*<0.01, ***= *p*<0.001. Rows in non-italicized text indicate measured values; rows in italics indicate derived values.

|  | **WT** | | **Cav-3OE** | |  |  |  |
| --- | --- | --- | --- | --- | --- | --- | --- |
|  | **3 mo** | **24 mo** | **3 mo** | **24 mo** |  |  |  |
|  |  |  |  |  |  |  |  |
| **Surface Sarcolemmal Membrane Area** |  |  |  |  |  |  |  |
| *From Intact (μm^2^)* | *18380 ± 898 (27)* | *21466 ± 1197 (24)* | *17119 ± 922 (24)* | *24562 ± 1003 (28) ###* | * |  |  |
| *From DT (μm^2^)* | *18502 ± 1191 (22)* | *18314 ± 518 (15)* | *18609 ± 736 (19)* | *22970 ± 824 (17) $$,##* | * |  |  |
| *DT/Intact* | *1.01* | *0.859* | *1.09* | *0.940* |  |  |  |
|  |  |  |  |  |  |  |  |
| ***I*_Ca_ density (pA/pF)** |  |  |  |  |  |  |  |
| Intact | -6.5 ± 0.4 | -4.6 ± 0.3 | -4.8 ± 0.4 | -5.2 ± 0.3 |  |  |  |
| Surface membrane | -2.2 ± 0.2 | -2.2 ± 0.2 | -2.7 ± 0.2 | -1.8 ± 0.2 |  |  |  |
| *T-tubule membrane* | *-14.6 ± 2.1* | *-7.1 ± 0.7 **** | *-9.2 ± 1.4* | *-9.3 ± 1* |  |  |  |
|  |  |  |  |  |  |  |  |
| ***I*_Ca_ density (pA/pF), adjusted for DT cell size** |  |  |  |  |  |  |  |
| Intact | -6.5 ± 0.4 | -4.6 ± 0.3 | -4.8 ± 0.4 | -5.2 ± 0.3 |  |  |  |
| *Surface membrane* | *-2.2 ± 0.2* | *-2.3 ± 0.2* | *-2.7 ± 0.2* | *-1.9 ± 0.2* |  |  |  |
| *T-tubule membrane* | *-14.3 ± 2* | *-8.2 ± 0.9 *** | *-8.1 ± 1.1* | *-10.1 ± 1.2* |  |  |  |
|  |  |  |  |  |  |  |  |

**Table S2.** Measured and calculated (italicized) cell and *I*_Ca_ parameters from ventricular myocytes isolated from 3- and 24-mo old WT and Cav-3OE mice. Data were subjected to *2*-*way* ANOVA and the results summarized in the right-hand columns, where “I” denotes an interaction between age and genotype and asterisks indicate significance. For cases where an interaction was significant, the results of Bonferroni *post-hoc* tests are included within the data columns: *, $ and # for comparisons with 3-mo WT, 3-mo Cav-3OE, or 24-mo WT cells, respectively. One, two, three and four symbols indicate *p*<0.05, <0.01, <0.001 and <0.0001, respectively. Differences in t-tubular *I*_Ca_ density were assessed using Student’s t-test where **= *p*<0.01, ***= *p*<0.001. Numbers in brackets indicate *n*/*N*.

|  | **WT** | | **Cav-3OE** | | **2-way ANOVA** | | |
| --- | --- | --- | --- | --- | --- | --- | --- |
|  | **3-mo** | **24-mo** | **3-mo** | **24-mo** | **I** | **OE** | **Age** |
|  |  |  |  |  |  |  |  |
| **Capacitance (pF)** |  |  |  |  |  |  |  |
| Intact | 217 ± 12  (27/10) | 269 ± 13  (25/6) ** | 203 ± 12 (25/8) | 315 ± 13  (28/7) $$$$, # | * |  | **** |
| DT | 152 ± 10  (22/6) | 146 ± 10  (16/3) | 146 ± 6  (19/5) | 180 ± 14  (18/6) $ | * |  |  |
|  |  |  |  |  |  |  |  |
| ***I*_Ca_ at 0 mV** |  |  |  |  |  |  |  |
| **Absolute (pA)** |  |  |  |  |  |  |  |
| Intact | -1380 ± 107 (27/10) | -1243 ± 85 (25/6) | -945 ± 70 (25/8) ** | -1608 ± 116 (28/7) $$$$, # | **** |  | ** |
| DT | -441 ± 39 (22/6) | -372 ± 20 (16/3) | -431 ± 34 (19/5) | -445 ± 44 (18/6) |  |  |  |
| **Density (pA/pF)** |  |  |  |  |  |  |  |
| Intact | -6.5 ± 0.4 (27/10) | -4.6 ± 0.3 (25/6) *** | -4.8 ± 0.4 (25/8) ** | -5.2 ± 0.3 (28/7) | *** | * |  |
| DT | -3.0 ± 0.3 (22/6) | -2.7 ± 0.2 (16/3) | -3.0 ± 0.3 (19/5) | -2.5 ± 0.2 (18/6) |  |  | * |
|  |  |  |  |  |  |  |  |
| ***T-tubule I_Ca_ density at 0 mV (pA/pF)*** |  |  |  |  |  |  |  |
| *T tubule membrane* | *-14.6 ± 2.1* | *-7.1 ± 0.7 **** | *-9.2 ± 1.4* | *-9.3 ± 1* |  |  |  |
| *T tubule membrane corrected for SA* | *-14.3 ± 2.0* | *-8.2 ± 0.9 *** | *-8.1 ± 1.1* | *-10.1 ± 1.2* |  |  |  |

**Table S3.** Steady-state Ca transients field-stimulated at 0.1, 0.2 and 1.0 Hz, as measured from ventricular myocytes isolated from 3- and 24-mo old WT and Cav-3OE mice. An asterisk indicates p<0.05 following a 2-way ANOVA, where “I” indicates an interaction between the two factors, however, a Bonferroni *post-hoc* test revealed no significant differences.

|  | **WT** | | **Cav-3OE** | | **2-way ANOVA** | | |
| --- | --- | --- | --- | --- | --- | --- | --- |
|  | **3-mo** | **24-mo** | **3-mo** | **24-mo** | **I** | **OE** | **Age** |
| *n/N* | 13/5 | 12/4 | 14/4 | 19/7 |  |  |  |
| **Amplitude (ΔF/F_0_)** |  |  |  |  |  |  |  |
| 0.1 Hz | 2.46 ± 0.25 | 2.27 ± 0.35 | 2.58 ± 0.30 | 2.16 ± 0.19 |  |  |  |
| 0.2 Hz | 2.54 ± 0.34 | 1.94 ± 0.35 | 2.17 ± 0.29 | 1.90 ± 0.16 |  |  |  |
| 1.0 Hz | 2.29 ± 0.38 | 1.82 ± 0.38 | 1.76 ± 0.26 | 1.63 ± 0.15 |  |  |  |
| **Time to peak (ms)** |  |  |  |  |  |  |  |
| 0.1 Hz | 40 ± 4 | 42 ± 6 | 51 ± 8 | 56 ± 8 |  |  |  |
| 0.2 Hz | 34 ± 4 | 40 ± 3 | 48 ± 7 | 55 ± 7 |  | * |  |
| 1.0 Hz | 30 ± 3 | 27 ± 3 | 31 ± 3 | 32 ± 2 |  |  |  |
| **Time to half decay (ms)** |  |  |  |  |  |  |  |
| 0.1 Hz | 365 ± 38 | 338 ± 40 | 304 ± 21 | 379 ± 26 | * |  |  |
| 0.2 Hz | 322 ± 36 | 332 ± 38 | 315 ± 27 | 370 ± 24 |  |  |  |
| 1.0 Hz | 217 ± 14 | 199 ± 17 | 206 ± 13 | 226 ± 10 |  |  |  |
